# Offering a tailored return to work program to cancer survivors with job loss: a process evaluation

**DOI:** 10.1186/s12889-016-3592-x

**Published:** 2016-09-06

**Authors:** M. P. van Egmond, S. F. A. Duijts, A. P. J. Scholten, A. J. van der Beek, J. R. Anema

**Affiliations:** 1 Department of Public and Occupational Health, EMGO+ Institute for Health and Care Research, VU University Medical Center, Van der Boechorststraat 7 – C581, 1081 BT Amsterdam, The Netherlands; 2 Research Center for Insurance Medicine, AMC-UMCG-UWV-VUmc, Amsterdam, The Netherlands; 3 Department of Psychosocial Research and Epidemiology, The Netherlands Cancer Institute, Amsterdam, The Netherlands

**Keywords:** Cancer, Intervention, Job loss, Occupational health care, Process evaluation, Return to work, Randomized controlled trial, Sick leave

## Abstract

**Background:**

In Europe, 1.7 million persons of working age are diagnosed with cancer each year. During or after treatment, cancer survivors (CSs) are vulnerable for job loss, and many CSs struggle with return to work (RTW). When offering RTW interventions to CSs, it is important to conduct a process evaluation to assess such factors as the population reached and implementation problems. Recently, we developed an innovative RTW program, tailored specifically to the needs of CSs with job loss in the Netherlands. The aim of this study was to evaluate the likelihood of theory and implementation failure, as well as to evaluate procedures for recruitment, execution and implementation of the tailored RTW program for CSs with job loss.

**Methods:**

Six components were evaluated in the RTW program: Recruitment, Reach, Dosage, Implementation, Satisfaction, and Experienced Barriers. Data were provided by logbooks and questionnaires from participating CSs, occupational health care (OHC) professionals, and re-integration coaches and job hunting officers who delivered the RTW program. SPSS and Excel were used to conduct the analyses.

**Results:**

85 CSs received the tailored RTW program. Their mean age was 47.9 years (SD 8.5). The majority were female (72 %), married (52 %), and of Dutch nationality (91 %). The program reached 88.2 % of the target population and 52 % of participants who started the program received the adequate dosage. The program implementation score was 45.9 %. Participants’ mean overall program duration remained within the protocol boundaries. Re-integration coaches were more satisfied with the program than job hunting officers or OHC professionals. Likewise, participants were more satisfied with the program delivery by the re-integration coaches than with the delivery by the job hunting officers. Reported barriers within the RTW program were a lack of communication, high program intensity and short program duration, and, with regard to the job hunting officers, a lack of experience with cancer-related RTW problems.

**Conclusions:**

Participants, OHC professionals, re-integration coaches and job hunting officers generally had positive experiences with the innovative tailored RTW program. Facilitating communication between the delivering parties, and engaging usual care during program delivery, could be key elements to improved program implementation.

**Trial registration:**

Dutch Trial Register, registration number NTR3562, registered 07-08-2012.

## Background

Each year, 3.45 million people are diagnosed with cancer in the European Union [1]. Of these, around half are persons of working age (aged between 15 and 64 years) [2]. The marked impact of cancer on workers has been documented by multiple studies [3–5]: in the first 6 years after diagnosis, between one quarter and half of cancer survivors (CSs) become unemployed [6, 7]. Across studies, CSs are 1.4 times more likely to become unemployed than healthy controls [8], and many CSs struggle with return to work (RTW) [6, 9].

A limited number of RTW interventions have been developed specifically for CSs [10, 11]. A review found 18 studies that offered re-integration interventions for CSs, of which three programs focused specifically on RTW [12]. From these studies, no definitive conclusions could be drawn with regard to the effectiveness of RTW programs for CSs. Also, the quality assessment of these studies revealed that the overall quality was low, and that study procedures should be improved in the future [12].

The first step to improving study procedures and program delivery is to evaluate the procedures of ongoing studies and programs, by conducting a process evaluation [13]. Process evaluations can be conducted alongside the delivery of intervention programs, and are aimed at assessing several process outcomes, such as the extent to which the target population was reached and the intervention was delivered according to protocol [14, 15]. Process evaluations allow researchers to better understand the individual intervention components, including their relation to each other, potential barriers to their implementation, and their impact on the intervention aims [13, 16, 17]. Further, process evaluations enable researchers to evaluate the likelihood of theory or implementation failure by linking the outcomes of the process evaluation to the effects of the program [15]. They also provide insight into the perceptions of the participants and stakeholders involved, and can contribute to the quality of future intervention studies. Moreover, the feasibility of, and incentives for, future implementation of an intervention program in daily practice can be identified through a process evaluation.

Recently, a tailored RTW program was delivered to sick-listed CSs with job loss in the Netherlands [18]. Previous studies have demonstrated that, for these CSs, RTW may be particularly challenging because of limited access to the labour market, the absence of opportunities for gradual RTW and workplace accommodations, and lack of support from an employer and colleagues. A tailored RTW intervention program could be an important step towards paid employment for these CSs [19]. Three organizations were contracted to deliver the program to the participants, and the program was implemented in cooperation with the Dutch Social Security Agency (SSA), as it is the agency with the primary legislative responsibility to support workers who lose their employment contract [20]. Given the multi-component character of the program, and the number of professionals involved [13], it was considered especially desirable to conduct a process evaluation alongside the tailored RTW program. Consequently, alongside the delivery of this program, data regarding process outcomes were collected.

This is one of the first studies in which an intervention for workers with job loss was developed, in cooperation with multiple organizations, and the occupational health services from the SSA. No previous studies of this kind were aimed at sick-listed workers due to cancer [21–23]. Therefore, the aim of this study was to gain insight into the feasibility of delivering the tailored RTW program to CSs with job loss in the Netherlands. Specifically, this study evaluated the procedures regarding recruitment, execution and implementation of the tailored RTW program, and evaluated the likelihood of theory and implementation failure. As the results with regard to the effectiveness of the program were not available at the time, this process evaluation will not link the program’s process outcomes to the effectiveness outcomes.

## Methods

### Design and procedures

This study concerns a process evaluation of the recruitment procedures, execution and implementation of a tailored RTW program for CSs with job loss in the Netherlands, which was offered within an experimental setting. The full study procedures and design of the RTW program have been published previously [18]. In summary, potentially eligible CSs with job loss were recruited for the RTW program from April 2013 to January 2015, by an invitation from the SSA. CSs who were interested in participating completed a screening questionnaire, after which the researchers contacted them by telephone to discuss participation. Those who were eligible to participate received a baseline questionnaire and informed consent form, and were included in the study after the completion and return of both. An information letter was sent to each participant’s general physician (GP) to inform them of their patient’s participation in the study, and to ask if there were any medical contra-indications for participation. If so, the researchers would deliberate with the GP whether participation in the program was appropriate. After enrolment in the study, CSs were randomly allocated to the intervention or control group. Participants in the intervention group received the tailored RTW program, as well as usual care provided by OHC professionals from the SSA.

### Tailored RTW program

**Fig. 1:**
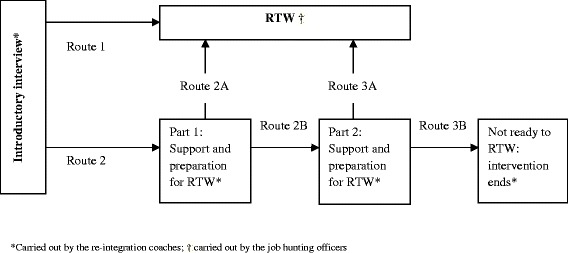
Design of the tailored RTW program

The tailored RTW program consisted of three parts: an introductory interview, a “Preparation for RTW” part, and a “RTW” part. The tailored RTW program encouraged participants to engage in developing a consensus-based RTW plan, to actively participate in coaching sessions to prepare for RTW, and to explore opportunities for RTW in therapeutic work or paid employment. The tailored aspect of the program was embedded in participants’ ability to select various routes in the program, which matched the individually required level of RTW support. The program is presented in Fig. 1.

For all participants, the tailored RTW program started with an introductory interview with a re-integration coach. In this interview, the coach and participant identified obstacles and possibilities for RTW. Drawing on this assessment, they chose a suitable route through the intervention program, by discussing if the participant was ready to “RTW” or if “Preparation for RTW” was needed. In addition, the participant’s expectations and present RTW activities were discussed. Participants who were already actively applying for jobs started with the “RTW” part of the program (Fig. 1: Route 1 in the program). In this part of the program, the coach would end the introductory interview by drawing up a short work profile, which included the participant’s wishes and capabilities for work. The work profile was used in the communication between the participant and one or two job hunting agencies. The job hunting agencies then attempted to place the participant in therapeutic or paid work that matched the participant’s wishes and capabilities. The staff at the two job hunting agencies that delivered the “RTW” part consisted mainly of vocational therapists or human resource experts. It should be mentioned that the job hunting agency staff had no specific expertise with regard to cancer. As the job hunting agencies had several locations across the Netherlands, participants travelled to the local office nearest to them for meetings with the agency.

For participants who concluded in their introductory interview that they were not involved in RTW activities (e.g., applying for jobs), the coach and participant decided that the participant would start with the “Preparation for RTW” part (Route 2 in the program). A re-integration agency, specialized in RTW coaching for CSs, delivered this part of the program (developing a RTW plan, coaching, and constructing a work profile). The coaches from this re-integration agency also conducted the introductory interviews at the start of the program. In general, these coaches were mostly former cancer patients who had experience with cancer survivorship and job loss. They had participated in training and education to become a RTW coach for cancer survivors. The meetings for the “Preparation for RTW” part of the program were held at the participant’s home or at a nearby office of the re-integration agency.

All program meetings between participants and re-integration coaches or job hunting agency personnel were face-to-face meetings. The program did not employ any peer-group activities. Alongside the “Preparation for RTW” part of the program, it was possible for participants to be referred to specialist physical or psychological care. This could happen if the participant and coaches concluded that the participant needed specialist care for such problems as extreme fatigue, psychological stress or trauma. Within the intervention program, the re-integration coaches registered when they referred participants to specialist care, but the content and duration of this care was not monitored as this was part of regular usual care within the Dutch healthcare system. The specific content of the program routes are described extensively below. The full content of the tailored RTW program was previously published as part of the study protocol [18].

#### Route 1

The coach and participant decided that the participant was ready to RTW. The coach contacted the researchers, who randomly assigned the participant to one of the two job hunting agencies (by using randomisation software). The participant and the selected agency held a meeting to explore job opportunities. According to the study protocol, the agency was expected to find at least two suitable jobs that matched the participants’ work profile, and their wishes for RTW. Further, these jobs could be either therapeutic or paid work, and had to be offered for at least three months. The protocol further dictated that the job hunting agencies should arrange for two job options within four weeks after the first meeting with the participant. If the agency was unable to meet these requirements, the second job hunting agency involved in this study joined the search for jobs.

#### Route 2

The coach and participant decided that the participant was not yet ready to RTW yet, and that (s)he needed preparation for RTW. In the following weeks, the participant and coach created a work profile, based on the participant’s wishes and needs for return to work. Also, the participant’s working experience and capabilities were taken into account. Alongside the development of the work profile, the re-integration coach held four to five individual coaching sessions with the participant. These sessions were scheduled to last for 1–1.5 h and were aimed at themes that the participant and the coach selected together. Within the protocol, eight predetermined themes were available, with the additional option to deviate from these themes if necessary. Examples of predetermined coaching themes were: “fatigue and managing energy levels and RTW”, “communication about cancer at work” and “stress, fluctuations in work ability and managing work, private life and recovery”. When the work profile and coaching sessions were completed, the participant and coach re-evaluated whether the participant was ready to RTW (Route 2A or Route 2B).

Route 2A: The coach and participant decided that it was time to RTW. The coach contacted the researchers, after which a job hunting officer was randomly assigned to the case of the participant. This route is similar to Route 1 (described above).

Route 2B: The coach and participant decided that the participant needed more preparation for RTW. The coach and participant held four to five additional coaching sessions focused on preferred themes. This process is similar to the process described in Route 2, with the exception that the work profile was completed at this stage. After participating in additional coaching sessions, the coach and participant re-evaluated if the participant was ready to RTW (Route 3A or Route 3B).

Route 3A: The coach and participant decided that the participant was ready to RTW. The coach contacted the researchers, after which a job hunting agency was randomly assigned to the participant’s case. This route is similar to Route 1 (described above).

Route 3B: It could be that the coach and the participant concluded that the participant was not ready to RTW after receiving the full “Preparation for RTW” part of the program. In that case, the intervention program was terminated and the participant’s case was referred to usual care for follow-up.

The maximum duration of the tailored RTW program was 7 months. The maximum duration of the “Preparation for RTW” part was 3 months; the maximum duration of the “RTW” part was also 3 months, and 1 month delay was calculated to allow for unforeseen events, such as illnesses or holidays. After participants completed the tailored RTW program, the researchers sent process evaluation questionnaires to the teams of OHC professionals, the coaches and the job hunting agencies. For participants, process evaluation questionnaires were sent 6 months after the start of their RTW program. Additionally, the researchers, as well as the re-integration coaches and job hunting officers who delivered the RTW program, kept logbooks on their activities and progress during program delivery.

### Target population

The target group for the tailored RTW program were CSs who were 18–60 years of age, had completed intensive cancer treatment, and were registered at the SSA as recipients of sickness or disability benefits due to cancer. CSs had to be sick-listed for a period of minimum 12 months and maximum 36 months. The 12-month cut-off value was chosen in accordance with the Dutch social security legislation, in which eligibility for benefits has to be re-evaluated after 12 months of sick leave. The limit of 36 months was chosen because data past 36 months of sick leave were not accessible at the SSA. The complete inclusion and exclusion criteria for participation in the RCT were previously published as part of the study protocol [18].

### Measures and data analysis

This process evaluation consisted of assessing six components within the tailored RTW program: Recruitment, Reach, Dosage, Implementation, Satisfaction, and Experienced Barriers. Relevant literature from Steckler and Linnan [15], as well as previously published frameworks, such as the RE-AIM (Reach, Effectiveness, Adoption, Implementation, and Maintenance) framework [24, 25], were taken into account in the design of this process evaluation. All reported results were based on available data: no measures of imputation were used to replace missing data. An overview of the process evaluation components is presented in Table 1. To analyze the data, we calculated descriptive statistics using Excel 2010 and SPSS 22.0 [26].

**Table 1:** Components of the process evaluation and data sources for evaluation

Components	Definition of the component	Data sources for component evaluation
Recruitment	Result of the recruitment procedures for participants	Research logbooks
Reach	Proportion of eligible participants who started participation in the tailored RTW program.	Research logbooks
Dosage	Flow diagram of proportion of chosen routes in the RTW program, and calculated adequate dosage of the program for participants. Frequency of chosen themes during coaching and additional referral to physical or psychological rehabilitation care.	Research and intervention logbooks
Implementation	Composite score of reach and dosage	Scores on reach and dosage
Satisfaction	Participants’ satisfaction with the content, intensity, and duration of the tailored RTW program, OHC professionals’ satisfaction of the use of the program along usual care; coaches’ and job hunting officers’ satisfaction in working with the program.	Questionnaires for participants, OHC professionals, coaches and job hunting officers
Experienced Barriers within the tailored RTW program	Summary of barriers in following or executing the RTW program as experienced by participants, coaches and job hunting officers. Reasons for not being referred to RTW.	Research and intervention logbooks, questionnaires, minutes from meetings

- Recruitment
  ‘Recruitment’ was defined as the result of all procedures to recruit eligible CSs for participation in the tailored RTW program. At the level of the participants, data regarding response were obtained from the research logbooks and displayed in a participant recruitment diagram. Recruitment was not evaluated at the organizational level (i.e., level of the OHC professionals, coaches and job hunting agencies), as the involvement of these organizations depended on the participant’s route through the intervention program.
- Reach
  ‘Reach’ was defined as the proportion of the target group that participated in the tailored RTW program. Participation in the program was defined as participating at least in the introductory interview, because this step was crucial as a starting point for each participant’s program. These data were provided by the research and intervention logbooks from the researchers and re-integration coaches.
- Dosage
  ‘Dosage’ was defined as the proportion of participants who started the program, who received an adequate dose of the tailored RTW program. Adequate dose was defined as having received a job offer through the services of the job hunting agency during the program. Participants who did not meet with the job hunting agency, because they had already found a job or decided to found their own company during the “preparation for RTW” part of the program, were also considered to have received an adequate dose.
  Further, the number of times each step in the RTW program was delivered, was described. Also, the mean overall duration of the RTW program, and range of duration between participants, was calculated. Additionally, it was reported if, and which, themes were discussed in the coaching sessions of the RTW program, and if the coaches referred participants to specialist physical or psychological rehabilitation care, alongside the RTW program. These data were provided by the intervention logbooks from the re-integration coaches and job hunting officers.
- Implementation
  ‘Implementation’ was defined as a composite score of the results of the components ‘reach’ and ‘dosage’, and was calculated by multiplying these proportions.
- Satisfaction
  ‘Satisfaction’ was defined as the extent to which the content, intensity, duration and delivery of the tailored RTW program was satisfactory according to the participants, OHC professionals, coaches and job hunting officers. Overall satisfaction and experience scores were calculated for each group. These data were captured by the process evaluation questionnaires. The OHC professionals, coaches and job hunting officers received one process evaluation questionnaire for each participant that was under their care.
- Experienced Barriers within the program
  This component summarized the experienced barriers with regard to participation in, or execution of, the tailored RTW program, as experienced by participants, OHC professionals, coaches and job hunting officers. These data were provided by the logbooks, process evaluation questionnaires, and by minutes from meetings between the researchers, the SSA, re-integration coaches and job hunting officers.

## Results

### Target population

The characteristics of participants in the tailored RTW program are described in Table 2. The recruitment of these participants was described as a component of the process evaluation (see below). The mean age of the participants was 47.9 years (SD 8.5). The majority were female (72 %), married (52 %), had children (65 %) and were of Dutch nationality (91 %). More than half of the participants were the principal wage earner of their household (54 %). Breast cancer was the most prevalent cancer among all participants (35 %).

**Table 2:** Baseline characteristics of CSs with job loss who participated in the intervention group of the RCT

Variable	Categories	Participants (*N* = 85)
		Mean (SD)
Age in years		47.9 (8.5)
		N (%)^a^
Gender	Male	24 (28)
	Female	61 (72)
Level of education	None/primary/lower vocational education	12 (14)
	Secondary school	18 (21)
	Vocational education/upper secondary school	32 (38)
	Upper vocational education/ university	23 (27)
Marital status	Living alone	17 (21)
	Married	43 (52)
	Living together	12 (15)
	Divorced/widowed	11 (13)
Having children	No	30 (35)
	Yes	55 (65)
(non-)Dutch nationality	Dutch	77 (91)
	Non-Dutch	8 (9)
Principal wage earner	No	39 (46)
	Yes	46 (54)
Type of sector previous job	Blue collar	7 (8)
	White collar	27 (33)
	Civil servant	26 (31)
	Health care worker	23 (28)
Type of employment contract prior to loss of employment	Fixed employment contract	25 (30)
	Temporary employment	47 (57)
	Temporary agency work contract	10 (12)
	Other type of contract	1 (1)
Previous job demands	Psychological and physical	27 (33)
	Mainly psychological	36 (43)
	Mainly physical	20 (24)
Tumor type	Breast	30 (35)
	Lung	1 (1)
	Gynecological	4 (5)
	Colon	10 (12)
	Gastro-intestinal	6 (7)
	Head and neck	2 (2)
	Skin/ melanoma	0 (0)
	Prostate	2 (2)
	Hematological	12 (14)
	Brain	1 (1)
	Other type of cancer	14 (17)
	Cancer recurrence	3 (4)
Treatment modalities	No treatment	2 (2)
	Surgery	64 (75)
	Radiotherapy	32 (38)
	Chemotherapy	52 (61)
	Hormone therapy	19 (25)
	Immunotherapy	8 (9)
	Other type of treatment	5 (6)
Declared free of disease	No	28 (33)
	Yes	57 (67)
Comorbidity	No	44 (52)
	Yes	41 (48)
		Mean (SD)
Work ability	(0–10)	4.7 (2.1)

^a^N and calculated percentages may approach or exceed the total N and 100 % because of missing values or rounding differences

### Components of the process evaluation

#### Recruitment

**Fig. 2:**
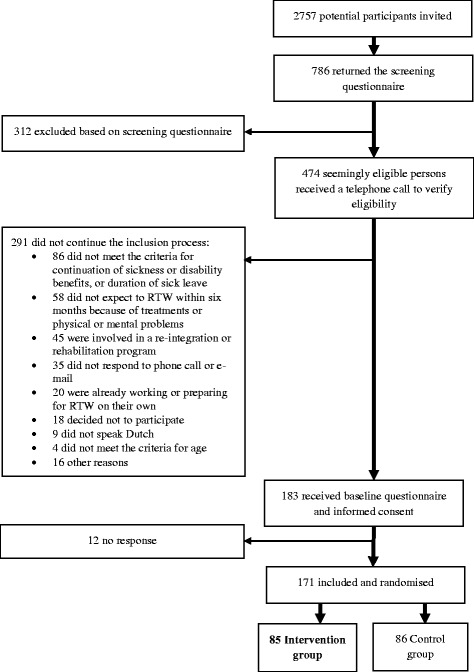
Participant recruitment diagram

In total, 2757 potential participants were invited to participate in the study, of whom 786 were interested in participating and returned the screening questionnaire. Among these, 312 did not meet the study inclusion criteria. The researchers contacted the remaining 474 potential participants by telephone. Of those, 291 did not continue the inclusion process due to various reasons: for example, 86 CSs did not meet the inclusion criteria for sickness or disability benefits and duration of sick leave, 58 CSs did not expect to be ready to RTW within six months, and 45 CSs were already involved or signed up for another re-integration or rehabilitation program. Also, 20 CSs had already returned to work or were in the process of RTW, and 35 CSs could not be reached. Of the 183 CSs who fulfilled the inclusion criteria and received a baseline questionnaire, 171 returned the questionnaire and were included in the study. After inclusion, none of the participants’ GPs reported a medical contra-indication for participation in the program. Of the 171 CSs in the study, 85 participants were randomly assigned to the tailored RTW program (Fig. 2).

#### Reach

Of the 85 CSs who were assigned to the tailored RTW program, 75 started the program by participating in the introductory interview. The other ten CSs did not start the program because of health problems and misinformation during recruitment regarding their eligibility for sickness or disability benefits. Therefore, the reach of the target group that participated in the tailored RTW program was 75 out of 85 CSs in the target group, i.e., 88.2 %.

#### Dosage

In Table 3, the six steps of the tailored RTW program are displayed, together with the proportion of participants who participated in each step. Of the 85 participants, 75 started the program, and 42 participants were referred to the job hunting agencies. In total, 33 participants were not referred to the job hunting agencies, for reasons that were reported as being work-related (14 participants), cancer-related (13 participants), personal (four participants), program-related (one participant) and unknown (one participant).

**Table 3:** Steps in the tailored RTW program

RTW program steps	Proportion of participants who received this step
	N (% of total 85 participants)
Step 1: Introductory interview	75 (88.2)
Step 2 (optional): Preparation for RTW (part 1)	54 (72.0)
Step 3 (optional): Preparation for RTW (part 2)	26 (34.7)
Step 4: Referral to job hunting agency for RTW	42 (49.4)
Step 5: Job hunting agency intake	41 (48.2)
Step 6: Job hunting agencies offered two suitable jobs	30 (35.3)

Specifically, regarding work-related reasons, nine persons had already found employment by themselves, or decided to found their own company. During the “Preparation for RTW” part of the program, three persons were not ready to return to work in their own perception, and two persons wanted to find a job without the assistance of the job hunting officers. Regarding cancer-related reasons, three persons were re-assessed at the SSA and found to be fully work disabled; seven persons suffered from medical complications and/or an increase in the level of physical and mental problems; two persons had recurrent cancer; and one person passed away during the program. Also, four participants had personal problems that, according to the re-integration coaches, prevented them from participating (fully) in the program, such as social, financial or psychological problems. Additionally, one person reportedly quit the program because (s)he did not agree with the approach taken by the re-integration coach. Afterwards, the coach reported that, in her perception, the participant had some personal problems which interfered with the participant’s ability to follow the program according to schedule. Attempts of the coach to stimulate the participant could have backfired, resulting in the fact that this particular participant quit the program.

To calculate the dosage, we assessed if jobs were offered to the participants by one or both of the job hunting agencies, which was the case for 30 participants, and if participants had already found employment, or decided to become an entrepreneur, before being transferred to a job hunting officer, which was true for nine participants. Ultimately 39 participants received the adequate dosage of the tailored RTW program (52 % of the 75 participants who started the program).

#### Themes during coaching sessions

For all 54 participants who participated in the program steps “Preparation for RTW (part 1 and optionally part 2)”, one or more themes were selected for the coaching sessions. Eight predefined themes were available during these steps in the program. The frequency of the chosen themes is presented in Table 4. The most popular themes were “Stress, fluctuations in work ability and managing work, private life and recovery” and “self-control, self-influence and resilience at work”. Both themes were chosen by 43 participants. The range of chosen themes between participants was zero to seven themes. Participants could choose different themes for each coaching session (with a maximum of eight themes). The median number of themes chosen per participant was five. Five participants did not choose any of the predefined themes, as it was also possible to deviate from the themes and discuss other personal challenges with regard to RTW.

**Table 4:** Chosen themes during the coaching sessions in steps “Preparation for RTW (part 1 and 2)”

Theme	N (% of 54 participants)
Introduction and planning of RTW program and coaching	2 (3.7)
Fatigue and managing energy levels and RTW	38 (70.4)
Cognitive and concentration problems and RTW	24 (44.4)
Stress, fluctuations in work ability and managing work, private life and recovery	43 (79.6)
Communication about cancer at work	28(51.9)
Self-control, self-influence and resilience at work	43 (79.6)
Increasing work ability and endurance in work and recovery	38 (70.4)
Legislation, rights, duties, and opportunities regarding work and illness	29 (53.7)

#### Referral to physical or psychological rehabilitation care alongside the program

Of the 54 participants in the program steps “Preparation for RTW (part 1 and optionally part 2)”, 27 participants were referred to rehabilitation care alongside the RTW program. Among these, nine were referred to physical care, nine to psychological care, and another nine participants were referred to both physical and psychological care. Participants receiving professional physical care were generally referred to (oncology) physical therapy (13 participants). Participants receiving psychological help were generally referred to a psychologist (12 participants).

#### Duration of the program

The protocol of the tailored RTW program allowed for a maximum program duration of 7 months, or 210 days. That is, 90 days were available for “Preparation for RTW (part 1 and optionally part 2)”, another 90 days were available for “RTW”, and 30 days were calculated for potential delays during the program. In practice, the mean duration of the tailored RTW program across participants was 156 days (SD 90), which was within time according to protocol. It should be noted that the overall duration also included participants who did not continue to the “RTW” part of the program. For participants who participated in both “Preparation for RTW (part 1 and optionally part 2)”, and “RTW”, i.e., the most extensive route in the program, the overall duration was 199 days (SD 84), which was still within protocol time.

Looking at the separate parts of the program, the mean duration of “Preparation for RTW (part 1 and optionally part 2)” was within the protocol deadline including delay, i.e., 117 days (SD 80). However, there was a large range in duration between the participants (7–373 days). The mean duration of “RTW” was 123 days (SD 59), which was longer than the protocol allowed, even with delay. Again, the range in duration of the RTW part was large (29–302 days).

#### Implementation

The implementation score was calculated by multiplying the percentage of the target group that was reached for the tailored RTW program, i.e., 88.2 %, with the percentage of participants who started the program, who received an adequate dosage of the program, i.e., 52 %. Therefore, the implementation score of the tailored RTW program was 45.9 %.

#### Satisfaction

Satisfaction was based on the process evaluation questionnaires. Of the 85 participants assigned to the RTW program, 68 participants returned the process evaluation questionnaire. Of those, five did not start participation in the RTW program. Their answers were therefore removed from the analysis.

For participants, satisfaction and experiences with the RTW program are presented in Table 5. In general, participants were more satisfied and reported more positive experiences with the “Preparation for RTW (part 1 and optionally part 2)” part of the program, than with the “RTW” part. The combined experience scores for “RTW” were actually slightly negative, i.e., 2.7 on a Likert scale of 1 to 5, with 1 being very dissatisfied and 5 being very satisfied. Participants thought the “Preparation for RTW (part 1 and optionally part 2)” part was more useful to their RTW and that the time trade-off (invested time versus returned benefits of the program) was better, compared to the “RTW” part of the program. Also, participants reported more confidence in the program delivery by the re-integration coaches, than that of the job hunting officers. Finally, the majority of participants who were offered employment through the job hunting officers reported feeling neutral to very dissatisfied in relation to the jobs they were placed in. Despite these ambiguity in experiences, over 70 % of participants would probably to certainly recommend the tailored RTW program to other CSs with job loss.

**Table 5:** Participants’ satisfaction with the tailored RTW program

Topics regarding the “Preparation for RTW 1 and 2” parts of the program	Participants (N=63) ^a^
Satisfaction (score range 1–5) ^b^	Mean (SD)
To what extent are you satisfied with:	
-Working with the re-integration coach	4.4 (0.8)
-Drawing up the RTW plan	4.2 (0.8)
-Program delivered by the re-integration coach (including themes)	4.2 (0.9)
-Drawing up a work profile in preparation for RTW	4.0 (1.0)
-Referral to a professional for physical rehabilitation care	4.2 (0.8)
-Referral to a professional for psychological rehabilitation care	3.9 (0.9)
Overall satisfaction score “Preparation for RTW part 1 and 2”	4.2 (0.2)
Experience statements “Preparation for RTW part 1 and 2” (score range 1–5) ^c^	Mean (SD)
-The RTW plan fit well with my wishes and needs for support	3.8 (1.0)
-The physical intensity of the program was all right	3.9 (1.0)
-The psychological intensity of the program was all right	3.8 (1.0)
-The duration of this part of the program was all right	3.3 (1.3)
Overall experience score “Preparation for RTW part 1 and 2”	3.7 (0.3)
Additional questions regarding steps “Preparation for RTW part 1 and 2”	N (%)
To what extent did you have confidence in the re-integration coach?	
-I had full confidence	27 (60.0)
-I had reasonable confidence	16 (35.6)
-I had little confidence	1 (2.2)
-I had no confidence	1 (2.2)
What did you think of the amount of time spent in this part of the program?	
-It was the right amount of time	27 (61.4)
-It took up a lot of time	5 (11.4)
-It didn’t take up a lot of time	12 (27.3)
What do you think of the amount of time invested and the returned benefits of participating in this part of the program?	
-It cost me little time and gained me a lot	21 (46.7)
-It cost me much time and gained me a lot	6 (13.3)
-It cost me little time and gained me little	13 (28.9)
-It cost me much time and gained me little	5 (11.1)
To what extent was it useful for you to participate in this part of the program?	
-Very useful	32 (71.1)
-Reasonably useful	9 (20.0)
-Neutral	1 (2.2)
-Not so useful	2 (4.4)
-Not at all useful	1 (2.2)
Topics regarding the “RTW” part of the program	
Experience statements (score range 1–5) ^c^	Mean (SD)
-The offered jobs fit well with my wishes and needs for RTW	2.9 (1.1)
-By working in the job that was offered, I feel that I can make it in the labour market	2.5 (1.1)
Combined experience score “RTW”	2.7 (0.3)
Additional questions regarding the RTW part of the program	N (%)
To what extent did you have confidence in the job hunting officers?	
-I had full confidence	6 (30.0)
-I had reasonable confidence	10 (50.0)
-I had little confidence	3 (15.0)
-I had no confidence	1 (5.0)
To what extent are you satisfied with working in the jobs offered:	
-Very satisfied	0 (0.0)
-Satisfied	2 (10.0)
-Neutral	12 (60.0)
-Dissatisfied	2 (10.0)
-Very dissatisfied	4 (20.0)
To what extent was the work easy to combine with other activities in your life?	
-It was easily combined	14 (70.0)
-It was not easily combined	6 (30.0)
What do you think of the amount of time invested and the returned benefits of participating in this part of the program?	
-It cost me little time and gained me a lot	1 (5.0)
-It cost me much time and gained me a lot	3 (15.0)
-It cost me little time and gained me little	8 (40.0)
-It cost me much time and gained me little	8 (40.0)
To what extent was it useful for you to participate in these steps of the program?	
-Very useful	4(20.0)
-Reasonably useful	4 (20.0)
-Neutral	8 (40.0)
-Not so useful	3 (15.0)
-Not at all useful	1 (5.0)
Overall, if you reflect on the complete RTW program, would you recommend this program to someone else in your situation?	
-Certainly	29 (49.2)
-Probably	14 (23.7)
-Maybe	10 (16.9)
-Unlikely	2 (3.4)
-Certainly not	4 (6.8)

^a^Total N may vary per question, as some parts of the program were optional, and some participants didn’t continue the program or did not return the questionnaire. Percentages for each question were calculated based on the number of participants that completed the question; ^b^A higher score reflects a higher level of satisfaction; ^c^A higher score reflects a higher level of agreement with the statement

Overall, the re-integration coaches reported the highest levels of satisfaction (3.8 on a Likert scale of 1-5), and the highest overall experience score regarding the execution of the tailored RTW program (4.4 on a Likert scale of 1-5) (Table 6). On the same scales, the OHC professionals reported the lowest levels of satisfaction (3.4) and experience (3.6) regarding the execution of the program. Further, 78.8 % of the re-integration coaches and 93.8 % of the job hunting officers thought that, in general, delivering the program increased their work load, but that they were not bothered by this.

**Table 6:** OHC professionals’, re-integration coaches’ and job hunting officers’ satisfaction with the tailored RTW program

Topics	OHC professionals (*N*=68)^a^	Re-integration coaches (*N*=52)^a^	Job hunting officers (*N*=48)^a^
Satisfaction (score range 1–5)^b^	Mean (SD)	Mean (SD)	Mean (SD)
To what extent are you satisfied with:			
-Protocol for delivering the program	N/A	3.8 (0.4)	3.7 (0.8)
-Instructions from my own organization	N/A	4.4 (0.5)	4.0 (0.5)
-Options to deviate within the program protocol	N/A	3.9 (0.6)	3.7 (1.0)
-Options for tailoring the program to participants’ needs	N/A	3.5 (0.7)	N/A
-Communication with a contact person within your organization	N/A	4.0 (0.6)	3.7 (0.6)
-Communication with the researchers	N/A	3.6 (0.5)	3.6 (0.7)
-Communication with the OHC professionals during the program	N/A	3.0 (0.8)	3.1 (0.8)
-Transfer from the re-integration coach to the job hunting officers	N/A	3.7 (1.1)	3.8 (0.6)
-Communication with the job hunting officers	N/A	3.3 (1.3)	N/A
-Communication with the re-integration coach	N/A	N/A	3.8 (0.5)
- Program completion and final contact with the participant	N/A	4.3 (0.6)	3.6 (0.8)
-General information about the program through the SSA	3.7 (1.0)	N/A	N/A
-Information about your patient participating in the program	3.6 (0.9)	N/A	N/A
-Opportunities to deliberate with the researchers	3.4 (0.8)	N/A	N/A
-Information regarding the content of your patients’ program	3.5 (0.9)	N/A	N/A
-Opportunities to deliberate with the re-integration coach	3.2 (1.0)	N/A	N/A
-Final report from the re-integration coach	3.5 (1.1)	N/A	N/A
-Information about your patients’ transfer to job hunting agencies	3.3 (1.0)	N/A	N/A
-Opportunities to deliberate with the job hunting officers	3.2 (1.0)	N/A	N/A
-Final report from the job hunting officers	3.4 (1.0)	N/A	N/A
Overall satisfaction score	3.4 (0.2)	3.8 (0.4)	3.7 (0.2)
Experience statements (score range 1–5)^c^	Mean (SD)	Mean (SD)	Mean (SD)
-The program fit well into my organization	3.7 (0.8)	4.5 (0.6)	4.4 (0.7)
-Before the program started, the program objective was clear to me	3.3 (1.0)	4.6 (0.5)	4.4 (0.6)
-Delivering the program was similar to my usual job demands	N/A	4.5 (0.6)	4.1 (1.0)
-Cooperating with the program agreed with my usual work tasks	3.7 (0.7)	N/A	N/A
-Before the program started, I was excited about it	N/A	4.7 (0.5)	4.6 (0.5)
-It was easy to follow the program protocol	N/A	3.9 (0.7)	3.5 (1.1)
-In hindsight, it was useful for me to participate in the program	N/A	4.2 (0.8)	4.2 (0.6)
-I was able to deliver my usual care alongside the program	3.6 (0.8)	N/A	N/A
-In the future, I would work with such a program again	3.8 (0.8)	4.6 (0.6)	4.5 (0.7)
Overall experience score	3.6 (0.2)	4.4 (0.3)	4.2 (0.4)
Time consumption of the program	N (%)	N (%)	N (%)
Delivering, or cooperating with, the program took up extra work time:			
-Yes and I did mind that	7 (10.3)	3 (5.8)	1 (2.1)
-Yes but I did not mind that	12 (17.6)	41 (78.8)	45 (93.8)
-Neutral	17 (25.0)	9 (15.4)	1 (2.1)
-No	29 (42.6)	0 (0.0)	1 (2.1)

^a^One process evaluation questionnaire was completed per participant, therefore, the N per group of professionals reflects the number of times a questionnaire was completed by a professional from that group. Also, due to missing values or rounding differences, N and percentages may approach or exceed the total N or 100 %; ^b^A higher score reflects a higher level of satisfaction; ^c^A higher score reflects a higher level of agreement with the statement

#### Experienced barriers within the program

Participants, re-integration coaches and job hunting officers reported that one of the main barriers within the RTW program was that the perceived duration of the program was too short, and that the perceived program intensity was too high. Specifically, some participants felt they needed more time to prepare for RTW, and they thought the “Preparation for RTW” part should be extended. Another important obstacle in the delivery of the program was the lack of clear communication between OHC professionals, re-integration coaches and job hunting officers. Specific barriers for communication among these parties were the large number of professionals involved and the fact that most communication went through digital channels (for feasibility reasons, as the program was offered on a national level). Also, the high workload of OHC professionals, and that a lot of participants’ files were frequently re-distributed within the SSA, e.g., when a new OHC team was assigned, or when the participant moved to a different district, contributed to these problems. As a result, some of the OHC professionals were not informed about the RTW program in an accurately or timely manner, and did not deliver the necessary documents to the re-integration coach. This delayed the start and progress of the RTW program for most of the participants.

Another obstacle was that, in the experience of participants and re-integration coaches, the job hunting officers had little experience with the health problems and the RTW process of CSs. As a result, the job hunting officers were not always able to intervene adequately in case of cancer-related problems. Further, participants and re-integration coaches reported specific problems in the delivery of the program by the job hunting officers, i.e., a lack of initiative and a lack of interest in the participants’ situation. To illustrate, it was reported during two meetings with the research team that the job hunting officers sometimes did not respond in time or at all to transfer requests by the re-integration coaches. Also, necessary documents from the job hunting officers were generally delivered late or not at all, and the documents provided to them by the re-integration coaches were often not used. The researchers requested that the job hunting officers use the documents from the re-integration agencies and to deliver their documents in time. However, there seemed to be a lack of motivation in the job hunting officers to do so. This led to delays in the delivery of the program. Further, at least two participants reported they had actually quit the program because they felt discouraged by the job hunting officers. For example, one participant mentioned that a job hunting officer had said that it would be very hard to find a job for him/her. A number of participants mentioned that they felt that the job hunting officers were only operating from a commercial perspective. As a result of these actions and the lack of clear communication, the re-integration coaches mentioned during the meetings that they had lost confidence in the “RTW” part of the program.

In contrast, many participants were very enthusiastic about program delivery by the re-integration coaches, and several participants gave them praise, such as that they felt they owed their new job to them. In two cases however, participants reported that the coach was not able to answer their questions, and that the program delivery was not person-oriented enough.

## Discussion

### Main results

The main results of this study are that the tailored RTW program reached 88.2 % of the target population, that more than half of the participants who started the program (52 %) received the adequate dosage, and that the implementation score was 45.9 %. The overall mean duration of the RTW program stayed within protocol boundaries; however, there was large variation between the participants in the program duration. Re-integration coaches reported the highest levels of satisfaction and positive experience with the program, compared to the job hunting officers or OHC professionals. Likewise, participants were more satisfied with the program delivery by the re-integration coaches than with the delivery by the job hunting officers. High program intensity and short program duration, as well as communication and cooperation problems, hindered the delivery of the RTW program.

### Interpretation of results

This was the first study to offer an innovative RTW program tailored to the needs of a specific subgroup of CSs, that is, CSs with job loss. In the Netherlands, previous studies were conducted that are, to some extent, comparable to the present study. For example, several RTW programs have been offered to sick-listed Dutch workers with distress, low back pain and musculoskeletal disorders [22, 27–29]. Generally, these studies proved that the implementation and execution of a RTW program for sick-listed workers is feasible. However, Lammerts et al. recently found that the implementation and execution of a participatory RTW intervention for workers with common mental disorders was less successful [30]. That study particularly reported obstacles in the implementation phase, as 28 % of the participants received a medical contra-indication for the program. In contrast, in the present study, no contra-indications for CSs’ participation in the program were reported. This may be explained by the strict and stepwise recruitment procedures of the present study, through which CSs with severe medical problems were eliminated from the inclusion process [18]. However, still thirteen participants in our study were unable to complete the RTW program because of physical and mental problems. Also, about one-third of the participants received additional physical or psychological rehabilitation care alongside the program. It could be that the participants took part in the additional rehabilitation care simply because it was offered to them, but it is also possible that for these participants, a contra-indication from the GP might have been appropriate. An explanation for the lack of reported contra-indications could then be that GPs lose contact with CSs during the cancer trajectory. That is, cancer trajectories can easily take up several months, during which the patient receives specialized medical care [31]. A previous study by Guassora et al. demonstrated that the transition between specialized care and primary care presents problems, and argued that GPs may need to be prepared to receive CSs in their daily practice [32]. It is also possible that the program information did not reach or fully inform some GPs, thereby limiting their assessment of the appropriateness of the program.

In the present study, the implementation score (45.9 %), as well as the number of participants who received the adequate dosage (52 %), was only moderately good. This was mainly the result of the fact that only 49.4 % of the participants was referred to the job hunting agencies. It should be mentioned that we did not set a specific goal for the program implementation score or the dosage, but it was implicitly expected that a higher implementation score and better dosage would be reached. Theory and/or implementation failure may explain these results. With regard to theory failure, it could be that the RTW program was only suited for a particular subgroup of the target group. That is, it could be that mainly relatively healthy CSs with job loss successfully participated in the RTW program [33]. The fact that several participants mentioned that the program was too intense and too short, and that some CSs with health problems could not continue the program, support this theory. In comparison, past RTW interventions for CSs had a longer duration than the program in the present study [12]. To illustrate, Stapelfeldt et al. offered a municipality-based RTW intervention for CSs in Denmark with a maximum duration of 1 year [34], and Tamminga et al. offered a hospital-based RTW intervention in the Netherlands with a maximum duration of 14 months [35]. It is worth considering that the duration or intensity of our program should be revisited. However, this would raise new questions with regard to the feasibility and financial aspects of the program [36]. In summary, we can hypothesize that there was indeed a mismatch between the target group and the RTW program, as not all CSs with job loss successfully participated in the program.

Further, we should consider that the present study may have suffered from implementation failure. For example, the duration of the RTW program varied greatly between participants, and several barriers for program delivery and participation were reported. An important barrier was the lack of clear communication between OHC professional, re-integration coaches and job hunting officers. A previous study by Anema et al. reported that cooperation and sharing information between OHC professionals and GPs can be problematic [37]. Perhaps this is also true for communication between OHC professionals, re-integration coaches and job hunting officers. Further, the program delay can be partly attributed to the recent economic recession in the Dutch labour market, which made it difficult for the job hunting officers to find employment for participants in this study [38]. Previous studies conducted in the past years reported similar delays in finding work opportunities for participants [22, 30].

Furthermore, there were specific indicators that the RTW program was not well implemented with the job hunting officers, i.e., their program delivery was delayed, there had been cooperation problems, and reportedly they had little specific knowledge with regard to cancer-related health problems. Specifically job hunting officers’ lack of sensitivity or experience with regard to cancer-related problems could explain the lower levels of satisfaction with their program delivery. In comparison, in the study by Tamminga et al., a RTW program was delivered by nurses with cancer expertise. In that study, participants reported high satisfaction scores [35]. It could be that the lack of knowledge and experience regarding cancer-related problems hindered the job hunting officers in delivering the program. It should also be considered that re-integration coaches who had unsatisfactory cooperation experiences with the job hunting officers, with whom they worked earlier in the intervention, willingly or unwillingly may have given a negative impression of the job hunting officers to participants who started the RTW program later on. As a result, participants may have decided against participating in the “RTW” part of the program, contributing to the moderate dosage and implementation scores.

An alternative explanation for the lack of transfers to the “RTW” part of the program could be that the “Preparation for RTW” part was quite successful. That is, the “Preparation for RTW” part was generally so well received by participants and professionals that some participants had already found employment and did not need the assistance of the job hunting officers anymore. This indicates that the “RTW” part of the program was in fact redundant for some participants.

### Strengths and limitations

The key strength of this study is that data were obtained through various sources (such as questionnaires and logbooks from all parties involved, and also minutes of meetings) in order to gain a full perspective on the process of delivering the RTW program. There are however several limitations to this study that should be mentioned. First of all only 63 out of 85 participants completed the process evaluation questionnaires. Further, in a few cases, data were missing on certain questions or dates, and the datasets for calculating satisfaction and experience scores were relatively small, especially for the job hunting officers, in which case the scores were based on 48 questionnaires. Additionally, we did not measure fidelity as a measure of the intervention program’s quality in this study. Because of the nature of the program, i.e., participants could choose their own route through the program, it was quite impossible to compare participants’ overall routes throughout the program. Therefore, we offered a comparison and evaluation of the program elements only, i.e., “Preparation for RTW” part and “RTW” part, instead of offering an overall interpretation on the program’s fidelity.

These factors limit the generalizability of our results. Also, the intervention logbooks from the job hunting officers were generally delivered past schedule, which could have introduced recall bias in the data. The qualitative results with regard to experienced program barriers should be interpreted cautiously, as they were based on individual comments. In order to gain a more comprehensive perspective of the participants’ experiences, a qualitative study inquiring about specific barriers in the program, could be conducted in the future. Also, the tailored RTW program was offered in cooperation with the Dutch SSA. Therefore, our results should be interpreted in the context of the Dutch social security system: the fact that the program delivery and implementation relied heavily on the social security context, could mean that efforts to replicate this study in another social or political context may be only partly successful.

### Implications for practice and research

This study demonstrated that, despite delays and several barriers in the program, CSs with job loss were generally satisfied with a RTW program tailored to their needs. The “Preparation for RTW” part of the program was the most appreciated element in the program. In order to prevent similar obstacles for program delivery in future studies, we would recommend that researchers introduce a pilot-phase in their studies, during which potential implementation problems can be identified and resolved [39]. As there seemed to be specific problems with the implementation of the program in the daily practice of the job hunting agencies, we would recommend that researchers specifically investigate the motives and capabilities of commercial parties when involving them in research projects, in order to ensure commitment and sustainable program delivery. Further, considering that long-term or permanent health problems are highly prevalent among CSs [40], we would recommend that experts delivering RTW programs to CSs receive training with regard to potential medical problems in the target group. For example, job hunting officers or other practitioners could participate in a seminar on cancer and work. Another aim of such training could be to enhance the quality and level of cooperation between these professionals. Previous studies in Denmark have demonstrated that satisfactory cooperation between groups of professionals can be difficult to achieve in a RTW context, not only for those working with CSs, but also for those working with patients with mental illness, for instance [41, 42]. Finally, we encourage clinical practitioners, OHC professionals and GPs to engage in future programs for sick-listed workers, for example by offering their expertise during recruitment [43]. This may facilitate a more accurate and efficient reach of the target population, and provides a guarantee for usual care to continue alongside intervention programs.

## Conclusions

In general, the participants, re-integration coaches, job hunting officers and OHC professionals had positive experiences with the innovative tailored RTW program. This program can be considered a first promising step towards tailored RTW support for CSs with job loss, and potentially for other sick-listed workers, with a significant challenge to labour market participation. Facilitating communication between the delivering parties, and engaging usual care during program delivery, could be key elements to improved program implementation.

## Abbreviations

CS(−s): Cancer survivor(−s)
GP: General physician
OHC: Occupational health care
RCT: Randomized controlled trial
RTW: Return to work
SSA: Social security agency
